# Editorial: Epigenetic Modifications Associated with Abiotic and Biotic Stresses in Plants: An Implication for Understanding Plant Evolution

**DOI:** 10.3389/fpls.2017.01983

**Published:** 2017-11-21

**Authors:** Mahmoud W. Yaish

**Affiliations:** Department of Biology, College of Science, Sultan Qaboos University, Muscat, Oman

**Keywords:** stress, epigenetics, evolution, molecular, plants, DNA methylation

Epigenetics in modern definition refers to the heritable alterations in gene expression which may lead to a variation in the phenotype without a change in the DNA sequence (Morris, 2001). Molecular events in epigenetics can take place naturally in cells but can also be modulated by environmental stressors. A combination of epigenetics and environmental stress dynamics lead scientists to investigate the impact of epigenetics on evolution in plants. The aim of the current research topic was to explore and update our understanding on epigenetic mechanism which may drive evolution in plants. The present edited volume includes original research and review articles describing epigenetic changes and their impact on the evolutionary adaptation mechanisms.

The adjustment of gene expression is a key mechanism used by plants during growth and developmental processes. Because plants are immobile organisms, the control of gene expression becomes more essential when plants are subjected to inescapable environmental stressors (Yaish et al., 2017). The expression level of a particular gene is manipulated by a series of coordinated epigenetic events on the nucleosomes, which involve DNA methylation, histone post-transitional modifications and small RNA interference (Baulcombe and Dean, 2014).

When exposed to stresses, plants tend to flower earlier than normal in order to skip the unfavorable conditions and produce seeds as soon as possible in order to conserve the species (Yaish et al., 2011). The resultant seeds may transfer the accumulated epigenetic information of the stressed plants to their progenies. Thus, this process may eventually lead to adaptive evolution. The transfer of epigenetic information over generations is called epigenetic transgenerational memory (Molinier et al., 2006). Such transgenerational epigenetic changes may be the result of heritable epialleles (Johannes et al., 2009), as well as through nucleosome recycling during cell division (Alabert et al., 2015). These findings are corroborated by the conclusions of the contribution by Iglesias and Cerdán that emphasized the essential role of nucleosome assembly on DNA replication processes and the stable epigenetic inheritance in plants. However, Iwasaki and Tricker, found in two other separate reviews published in this topic that the changes in chromatin can also be reverted to the default standard in order to reduce potentially negative impacts on the respective phenotype. On the other hand, Latzel et al. showed that epigenetic memory could be an essential motor which empowers environmental adaptation or intelligent behavior of clonal plants.

The relationship between DNA methylation and gene expression is not direct, and not yet completely understood (Law and Jacobsen, 2010), partially due to the involvement of other epigenetic factors in controlling gene expression. Small non-coding RNA sequences (sRNA), generated by the epigenetic machinery of cells, can alter gene expression or even block the translation of the targeted mRNA in the ribosomes (Carrington and Ambros, 2003). Bioinformatic analysis work carried out by Yakovlev and Fossdal, have confirmed that sRNA can play an important role in the establishment of epigenetic memory in Norway spruce. Another study published in this topic has shown that there is a crosstalk mechanism between DNA methylation and sRNA expression in *Populus siminii* plants when exposed to temperature stress; thus, DNA methylation influences miRNA expression and, in turn, may regulate the expression of their natural mRNA targets (Ci et al.). Additionally, Matsunaga et al. showed that sRNA has the ability to regulate the expression of the Ty1/copia retrotransposon, ONSEN, when Arabidopsis is grown under heat stress. It is worth saying here that different epigenetic players can be involved in regulating a tolerance mechanism in plants. For example, Liu et al. highlighted in their review the critical regulatory role of different epigenetic players including DNA methylation, histone modifications and sRNA in modulating the expression of thermotolerant genes, a complicated mechanism, which requires further investigation. Interestingly, the contribution by Espinas et al. revealed that transgenerational transfer of pathogen defense signaling and priming is also controlled by epigenetic mechanisms similar to those found in plants, due to abiotic stresses. In addition, Han and Luan illustrated in their review that sRNA species can be horizontally transferred from plants to mammals and from microbes to plants, and these cross-kingdoms sRNA molecules are important in host gene silencing mechanisms. Horizontal movement of sRNA may represent an additional mechanism of plant evolution through epigenetics.

Environmental stresses on some plant species lead to DNA methylation/demethylation (Yaish, 2013; Al-Lawati et al., 2016). These changes can be maintained and transmitted to the offspring, even in the subsequent absence of the original stress however, the DNA methylation alteration is not constant among the different stress episodes and plant species. For example, Eichten and Springer have proven that DNA methylation patterns in corn are lacking consistency when the plants are subjected to cold, heat and ultraviolet irradiation. Therefore, the inheritance of changes in DNA methylation in corn, possibly related to changes in phenotypes, is probably not robust. However, in other experiments, DNA methylation has shown some consistency in terms of heritability. For example, methyl-sensitive amplified fragment polymorphism analysis has revealed that DNA methylation, when induced by laser irradiation, is heritable and has induced the expression of miniature inverted-repeat transposable elements (MITEs) in rice (Li et al.).

The Arabidopsis histone H3K4 methyltransferase PRDM9 was shown to exert a clear effect on the distribution of meiotic recombination hotspots (Choi et al., 2013). DNA methylation patterns may also affect chromosome recombination events during meiosis in plants. Recent studies have shown that DNA hypomethylation in Arabidopsis, due to the loss of function in DNA methyl transferase (*MET1*), leads to alterations in the distribution of the crossover events throughout the chromosomes during mitotic cell division (Mirouze et al., 2012; Yelina et al., 2012). Given this notion, DNA methylation will theoretically affect the production of new alleles and eventually the appearance of new phenotypes and species, a process that could promote evolution in plants. However, recent work on genomes of different plant species revealed that the evolutionary factors that control changes in DNA methylation significantly vary across plant species, genes and methylated sequence contexts (Takuno et al., 2016).

The issues being raised here concern how far we are from decoding the evolutionary mechanism based on epigenetics and whether stress facilitates or accelerates the appearance of new species. Since the days of Lamarck (1744–1829 AD), who introduced the theory of the heritability of acquired characters, scientists tried unsuccessfully to prove that epigenetic inheritance is the reason behind the appearance of new phenotypes under stress conditions (Pigliucci and Finkelman, 2014). The situation has not changed much, even after recent discoveries in molecular epigenetics, which indeed reveal some evidence on the fluctuating relationship between environmental stresses and the appearance of novel phenotypes.

The use of artificially induced methylation changes led to the establishment of stable epialleles measurable as epiQTLs (Cortijo et al., 2014), and epigenetic recombinant inbred lines (epiRILs) (Johannes et al., 2009). These set-ups permitted to test the appearance, amongst these libraries, of phenotypic variants, thereby establishing correlations between phenotype and epigenotype. However, this example in Arabidopsis cannot be generalized to all plant species growing under natural conditions. The difficulties in considering epimutations in adaptive evolution processes stem from the fact that deciphering the effects of epigenetic alterations on gene expression and DNA recombination frequency is complex and even elusive.

Epigenetic variations are basically controlled by DNA-coded chromatin modifier proteins. This point was emphasized when Ding and Mou studied the ELONGATOR, multiprotein complex involved in epigenetic regulation in animals and plants, and found that this protein plays a role in histone acetylation and DNA methylation in plants. Consequently, mutations within the corresponding genes led to abnormal growth and development, and deviant susceptibility to various stressors in *Arabidopsis thaliana*. Similarly, in another review article, Moraga and Aquea have concluded that members of the Spt-Ada-Gcn5 ACETYLTRANSFERASE (SAGA) protein family complexes may regulate gene expression under various stress conditions in plants through the posttranslational modification of some histones. Therefore, epigenetic changes are not independent agents of adaptation and evolution; rather, they can mediate the mechanism that controls this process (Kronholm, 2017).

In conclusion, research that seeks to understand epigenetic mechanisms and their relation to stress adaptation may help, to a certain degree, to appreciate the evolution scenarios of some species under specific conditions; however, the information yielded by different branches of epigenetic research is unable to provide epimutations with a clear role in evolutionary adaptation mechanisms. This fact suggests the presence of other unknown epigenetic players, which control this process and, thus, clearly warrant more in-depth investigations.

## Author contributions

The author confirms being the sole contributor of this work and approved it for publication.

### Conflict of interest statement

The author declares that the research was conducted in the absence of any commercial or financial relationships that could be construed as a potential conflict of interest.
